# Construct Validation of Self-Determination Theory in Second Language Scale: The Bifactor Exploratory Structural Equation Modeling Approach

**DOI:** 10.3389/fpsyg.2021.732016

**Published:** 2021-09-30

**Authors:** Abdullah Alamer

**Affiliations:** ^1^Department of English, Imam Muhammad Ibn Saud Islamic University, Al-Ahsa, Saudi Arabia; ^2^Department of English, King Faisal University, Al-Ahsa, Saudi Arabia

**Keywords:** construct validation, bifactor exploratory structural equation modeling, convergent validity, discriminant validity, langauge learning, exploratory structural equation modeling (ESEM), confirmatory factor analyses (CFA), self-determination theory (SDT)

## Abstract

The present study aimed to assess the construct validity of the Self-Determination Theory in Second Language Scale (SDT-L2; Alamer, 2021). The study involved 266 undergraduate students learning English as a second language (L2) in Saudi Arabia. The factorial structure of the SDT-L2 was examined using the advanced bifactor-Exploratory Structural Equation Modeling (ESEM) method. The scale provided adequate composite reliability and the bifactor ESEM provided unique details about the multidimensionality of the scale which accounted for the specific constructs (i.e., intrinsic, identified, introjected, and external regulations) and the general constructs (i.e., autonomous motivation and controlled motivation), and allowed for assessment of convergent and discriminant validity. Predictive validity was established by showing that autonomous motivation significantly predicted L2 performance, while controlled motivation did not. Overall, the study demonstrated the usefulness of the bifactor ESEM for construct validation purposes and the results showed that SDT-L2 is a valid scale to assess students’ L2 motivation based on SDT perspective.

## Introduction

Self-determination theory (SDT) is a macro theory of motivation that has been applied to several life domains to understand what drives individuals to pursue their goals (Ryan and Deci, 2000, 2020). SDT has demonstrated valuable and valid insights about the motivation of learners who learn a second language (L2) in different language contexts (Noels et al., 1999; Alamer, 2021; Alamer and Almulhim, 2021). Essentially, SDT posits that motivation is multidimensional and depends on the extent to which individuals originate their behavior from within (Ryan and Deci, 2000). The theory maintains the existence of two general types of motivation, *autonomous motivation*, and *controlled motivation* with each having two sub-types of regulations. Autonomous motivation refers to the quality of individuals’ motivation being volitional. At the extreme of autonomous motivation is *intrinsic regulation* which reflects language learners’ inherent inclination toward carrying out the language tasks. Next comes *identified regulation* which is seen as an extrinsic type of autonomous motivation, and reflects the value and importance language learners attach when carrying out the language tasks. In contrast, controlled motivation refers to the type of motivation that is controlled by external circumstances and has two sub-types of regulations. At the extreme of controlled motivation is *external regulation* which reflects language learners’ desire to get rewards or avoid punishments in carrying out language tasks. Next comes *introjected regulation* which is seen and partially internalized into the self (i.e., less controlled) and reflects the internal pressure language learners have in carrying out language tasks such as avoiding shame, guilt for failing, or anxiety. In the L2 domain, researchers assessed the relation between these regulations and different language outcomes including willingness to communicate (Watanabe, 2011), engagement (Oga-Baldwin and Nakata, 2017; Dincer et al., 2019; Noels et al., 2019), positive affect (Alamer and Lee, 2019), students’ GPA (Noels et al., 1999), and attainment of the vocabulary (Alamer, 2021). These studies confirmed the positive relationships between the more autonomous types of motivation and the outcomes while showing that controlled types of motivation are either unrelated or negatively related to the outcomes.

One of the earliest scales presented to the field that assesses SDT regulations is perhaps Noels et al. (1999) scale. Based on the SDT literature, the researchers used correlation analysis to provide preliminary evidence of scale reliability and validity. The field has benefited from using this scale for different research contexts, but recent research showed that there is room for improvement. For example, Alamer and Lee (2019) pointed out that introjected regulation consistently results in weak reliability in the literature (Cronbach’s alpha (α) as low as 0.59) because the construct is measured by only two items. Collier (2020) indicated that factors in CFA that are assessed by only two items usually yield measurement issues in the solution including weak reliability estimates. Although Noels et al. (1999) scale has gone through standard CFA (Ardasheva et al., 2012), its reliability estimates were rather low (again, as low as 0.58 in three distinct samples) and the correlation between the constructs was inflated because of the constraints CFA imposes. Critically, the correlation between intrinsic regulation and external regulation was positive and large (*r* = 0.87) which has been increased substantially from (*r* = 0.55) in the EFA (i.e., Δ*r* = 0.32), thus the CFA results contradict the SDT distinction between the two constructs. Further, the formulation of SDT postulated the co-existence of two overarching constructs (i.e., autonomous motivation vs. controlled motivation) with each having two sub-types of regulations. This formulation cannot be captured statistically by correlation or the typical Exploratory Factor Analysis (EFA) or standard CFA because they do not account for the assessment of global constructs. For these reasons, Alamer (2021) has developed a modified scale, named SDT-L2 scale, based on the work of Noels et al. (1999) and presented preliminary evidence of the construct validity. The SDT-L2 scale has four subscales representing the four regulations with each having 5 items equally. Alamer (2021) evaluated the construct validity and reliability of the scale by using higher-order CFA. The higher-order model was chosen because it permits the inclusion of second-order factors that affects first-order factors (e.g., autonomous motivation as a second-order factor affecting the first-order factors, intrinsic and identified regulations). The analysis provided satisfactory results and the study is perhaps the first to account for the global constructs of autonomous motivation and controlled motivation in a measurement model.

**FIGURE 1:**
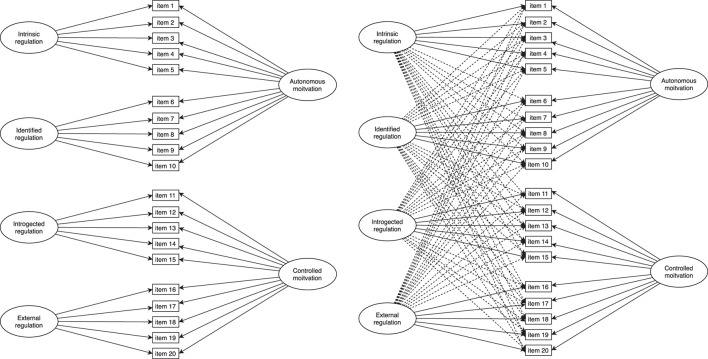
Visual representation of the bifactor CFA (on the left) and bifactor ESEM (on the right).

Nonetheless, recent advancement in construct assessment has brought a relatively new method called Exploratory Structural Equation Modeling (ESEM) that integrates the best of CFA and EFA in one analysis (Marsh et al., 2009; Morin et al., 2013; Alamer and Marsh, 2022). ESEM is similar to EFA as it allows items to cross-load on all factors involved in the analysis and differs from the EFA as it takes on the features of Structural Equation Modeling (SEM) such as evaluating model fit indices, assessing and allowing to correlate the measurement errors, and allowing for different model specifications to be included [see Alamer and Marsh (2022) who introduced the method to the L2 field]. In almost all multidimensional studies, ESEM presents a better fit to the data because of the flexibility and less restrictive system it has over the CFA (see Morin et al., 2016 for details). Within the CFA and ESEM frameworks, a bifactor model can be evaluated (Reise et al., 2010). Bifactor models are alternatives to the higher-order models which postulate the co-existence of the general factors along with specific factors (Morin et al., 2016). In bifactor CFA, the items are loaded on their specific factors and on the general factors they presume to correspond to, while in bifactor ESEM all items loaded on the specific factors while loading on the general factors they presume to represent. In both bifactor CFA and ESEM, the factors are set to be orthogonal (i.e., correlations are set to be zero). Figure 1 illustrates visually the difference between the two models. Howard et al. (2018) state that bifactor models should be selected over the higher-order models unless strong conceptual justifications are present. Thus, we take on this perspective in assessing the construct validity of SDT-L2 scale.

Multidimensional research often reported that bifactor models (whether in ESEM or CFA) provide a better model fit to the data and capture the co-existence of the global (general) factor meaningfully (Reise et al., 2010; Morin et al., 2016). This is true with studies that evaluate the SDT subscales, wherein researchers observe that bifactor models are superior to the standard solutions. For example, Howard et al. (2018) has investigated the multidimensionality of SDT scale for workplace among Canadian employees and tested its continuum structure. The researchers achieved this by contrasting the results of bifactor CFA with those of bifactor ESEM and the findings showed that the bifactor CFA solution did not fit the data appropriately whereas the bifactor ESEM did. The researchers then evaluated a structural bifactor ESEM to examine how the factors are related to outcomes such as employees’ positive affect, and the results were in line with the SDT proposition. The authors concluded that bifactor ESEM “provides an alternative approach that allows for the simultaneous consideration of the global quantity of self-determined motivation together with all qualitative variations along the SDT continuum in a single model not tainted by multicollinearity” (Howard et al., 2018, p. 22).

The SDT-L2 scale (Alamer, 2021) has been successfully introduced to the language learning field to assess L2 learners’ motivation based on the SDT (Alamer and Lee, 2021), however, the internal structure of this scale has not been replicated using the more appropriate statistical tool such as the bifactor ESEM to capture the appearance of the global factors (autonomous motivation and controlled motivation). Thus, the present study aimed to replicate the construct validity and predictive validity of the SDT-L2 scale in a more appropriate manner. To do so, the author first compare the results of the bifactor CFA and bifactor ESEM in terms of goodness-of-fit. Second, the author proceed with the solution that shows a better fit and tests its predictive validity for language outcome (i.e., L2 performance). Based on previous studies (e.g., Howard et al., 2018), it is hypothesized that bifactor ESEM would fit the data better and would be able to capture the variance in both specific and global factors meaningfully. In addition, as it is theoretically and empirically supported (Alamer and Lee, 2019; Dincer et al., 2019; Noels et al., 2019), it is postulated that in the structural bifactor ESEM, autonomous motivation would positively predict scores in L2 performance, while controlled motivation would be negatively or unrelated to L2 performance.

## Methods

The study sample consisted of 266 Saudi undergraduate students who learn English as the L2 in Saudi universities. The participants were aged between 18 and 22 years, with a mean age of 19.3 years (SD_age_ = 0.46). The sample was 42% male and 58% female. A convenience sample strategy was used, and students were invited through email to participate in an online questionnaire made by Google Forms. Students who do not want to participate or want to withdraw while participating were asked to refrain from completing the questionnaire and simply close the webpage.

### Measures

#### The Self-Determination Theory in Second Language Scale

The SDT-L2 scale comprises 20 items measuring 4 specific constructs of SDT, intrinsic regulation, identified regulation, introjected regulation, and external regulation (see the Supplementary Appendix for the full scale). The scale was designed in a 5-point Likert-type response format. Each subscale consists of five items [and the four subscales represent the Two overarching constructs (i.e., autonomous vs. controlled)]. Participants Were asked to ponder the question “Why Are you learning English?” and then indicate the extent to which they agreed With the statements that followed. Example items Are as follows: For intrinsic regulation, “because I enjoy learning English”; For identified regulation, “because learning English Is important for my personal growth”; For introjected regulation, “because I Would feel ashamed if I am Not successful in English learning like my friend (s)/family”; And for external regulation, “because I just want to pass the English exam.”

#### L2 Performance

Students’ language performance was assessed using two measures. The first is their students’ grade point averages (GPA) in their English courses. Students’ scores on this measure ranged from 1 to 5, with 5 representing excellent progress in the language courses. The units of English lessons include subjects of Reading, Writing, Speaking, Listening, Vocabulary, and Phonetics. The second measure was students’ effort in learning the L2. Five items taken from Gardner’s (2010) measure of effort were used. An example item is “I really work hard to learn English.”

#### Statistical Analyses

To assess the validity and reliability of the SDT-L2 scale, The author used two software tools: Jamovi (The Jamovi Project, 2019) and Mplus 8.1 (Muthén and Muthén, 1998). First, the author tested univariate normality by inspecting the skewness and kurtosis using the “+2/−2” and +10/−10 guidelines, respectively (Collier, 2020). The author obtained the reliability of the constructs using composite reliability (CR). CR (also called coefficient omega) considers the factor loadings of the observed variables and their measurement errors in the calculation. Hence, this reliability measure is obtained from the measurement model (see Collier, 2020 for details). To test the goodness-of-fit, the χ^2^ and its *p*-value were evaluated. Alternative measures were also used such as the comparative fit index (CFI), the Tucker-Lewis index (TLI), the root mean square error of approximation (RMSEA), and the standardized root mean square residual (SRMR) were used. CFI and TLI values that are in the region of 0.95 are indicative of a good model fit. Values smaller than 0.08 or 0.06 for the RMSEA and SRMR support, respectively, acceptable and good model fit (Hu and Bentler, 1999; Marsh et al., 2004). Convergent validity was assessed by inspecting the strength of items loading on their respective factors in the ESEM solution and discriminant validity was tested by showing that items presumed to load on one factor do not load on the others in the ESEM (Guay et al., 2015). In both bifactor CFA and bifactor ESEM, the maximum likelihood estimation with robust standard errors (MLR) is used.

## Results

The results shown in Table 1 indicate that the data have a relatively normal distribution. In addition, the correlation patterns seem to be within the expected direction. The CR values are reported in Table 2 based on the bifactor CFA and bifactor ESEM solutions.

**TABLE 1:** Descriptive statistics and zero-order correlations for the variables.

Variable	*M*	*SD*	Skew/Kurtosis	1	2	3	4	5	6
*1. GPA*	4.07	1.12	−0.31/−0.18	—					
2. Effort	3.75	1.17	−0.25/−0.16	0.36***	—				
3. Intrinsic regulation	3.66	0.89	−1.56/2.34	0.35***	0.47***	—			
4. Identified regulation	4.17	0.90	−1.87/3.19	0.31***	0.42***	0.69***	—		
5. Introjected regulation	3.38	1.34	−0.42/−1.12	−0.18**	0.01	−0.03	0.17*	—	
6. External regulation	4.23	0.86	−1.01/-0.07	−0.11	−0.18**	0.05	0.07	0.38***	—

*p < 0.05, **p < 0.01, ***p < 0.001 Examining the Factor Structure of the SDT-L2.

**TABLE 2:** Bifactor CFA and bifactor ESEM factor loadings of the SDT-L2 scale.

Items	Bifactor CFA			Bifactor ESEM					
	Autonomous	Controlled	Specific factor	Autonomous	Controlled	Intrinsic	Identified	Introjected	External
Intrinsic 1	0.82*		0.54*	0.80*		0.26*	0.02	0.05	−0.17*
Intrinsic 2	0.59*		0.61*	0.77*		0.52*	0.14	0.02	0.05
Intrinsic 3	0.75*		0.56*	0.80*		0.43*	0.07	0.07	–0.16
Intrinsic 4	0.69*		0.71*	0.72*		0.21*	0.06	–0.06	0.04
Intrinsic 5	0.79*		0.76* (0.85)	0.83*		0.19	0.03	0.13	–0.05
Identified 1	0.81*		0.63*	0.72*		0.11	0.34*	–0.01	0.03
Identified 2	0.69*		0.58*	0.63*		0.18	0.25*	–0.07	–0.06
Identified 3	0.74*		0.56*	0.71*		0.06	0.60*	0.05	–0.03
Identified 4	0.77*		0.52*	0.68*		0.01	0.65*	–0.03	0.06
Identified 5	0.69*		0.53* (0.89)	0.64*		0.09	0.56*	–0.02	–0.08
Introjected 1		0.14	0.75*		0.18	0.03	0.31*	0.66*	0.09
Introjected 2		0.25*	0.75*		0.22*	0.15	0.15	0.83*	0.04
Introjected 3		0.13	0.66*		0.18	–0.04	–0.03	0.43*	0.16
Introjected 4		0.28*	0.62*		0.36*	0.06	–0.02	0.07	–0.04
Introjected 5		0.02	0.62* (0.67)		0.01	–0.08	0.06	0.41*	0.28*
External 1		0.32*	0.80*		0.22*	–0.09	0.09	0.09	0.80*
External 2		0.41*	0.88*		0.43*	–0.01	–0.04	0.02	0.92*
External 3		0.13	0.62*		0.35*	–0.07	–0.07	0.32*	0.37*
External 4		0.42*	0.61*		0.70*	–0.04	–0.01	0.43*	0.16
External 5		0.41*	0.63* (0.74)		0.43*	0.08	–0.04	0.12	0.62*
CR	0.84	0.82		0.81	0.70	0.50	0.38	0.57	0.48

*p < 0.05; CR values for the bifactor CFA specific factors are in parentheses.

The results of initial bifactor CFA and bifactor ESEM results in convergence issues of the solutions. The modification indices suggested that errors in two items on intrinsic orientation (item 1 and item 2) are highly correlated. The two items seem to be quite similar in wording, thus they appear to share similar measurement errors. After correlating the error terms, the two models converged. It appeared that the bifactor CFA yielded less than optimal fit to the data [i.e., χ^2^(125) = 334.516, *p* < 0.05, CFI = 0.91, TLI = 0.88, RMSEA [90% CI] = 0.09 [0.08; 0.10], SRMR = 0.07]. In contrast, the bifactor ESEM appeared to fit the data well [i.e., χ^2^(94) = 204.798, *p* < 0.05, CFI = 0.96, TLI = 0.91, RMSEA [90% CI] = 0.07 [0.06; 0.09], SRMR = 0.03].

Although the bifactor ESEM outperforms its counterpart bifactor CFA, it was believed appropriate to provide the factor loadings of the two models for the sake of empirical comparison. As it can be seen in Table 2, the factor loadings in both solutions appear to reflect the idea of SDT theoretical underpinning. A key advantage of the bifactor ESEM, in addition to its better fit to the data, is that it accounts for the general factors adequately (i.e., autonomous motivation and controlled motivation) over the variance that already expressed through the specific factors (intrinsic, identified, introjected, and external regulations). That is, the loadings on the general factors were high and positive for the items related to its presumed general factors (for autonomous motivation the loadings ranged from 0.63 to 0.83, and for controlled motivation, the loadings ranged from 0.01 to 0.70). Moreover, it appears that items on intrinsic and identified regulations loaded more strongly on the general factor “autonomous motivation” than on the specific factors. Except for one item (i.e., introjected 4) the items of “introjected regulation” loaded strongly on their specific factor. A similar observation is found among the items of “external regulation” in which only the loading of “external 4” was stronger on the general factor “controlled motivation” than on the specific factor.

Accordingly, we proceed with the bifactor ESEM to assess the predictive power of the SDT-L2 scale for the outcome. In the structural bifactor ESEM, the latent variable “L2 performance” was included as a dependent latent variable to examine the extent to which the two general factors (i.e., autonomous motivation and controlled motivation) predict the outcome as hypothesized. It should be noted that a bifactor ESEM where both the general and specific factors allowed to predict the outcome resulted in a non-converged solution. Therefore, the author retain a bifactor ESEM model where the outcome was only predicted by the general factors. Table 3 shows that this model has yielded good model fit [i.e., χ^2^(131) = 265.722, *p* < 0.05, CFI = 0.94, TLI = 0.90, RMSEA [90% CI] = 0.07 [0.06; 0.09], SRMR = 0.05]. The path coefficients of this model indicates that autonomous motivation strongly and positively predicted L2 performance (β = 0.87, *p* < 0.001), while controlled motivation failed to predict L2 performance (β = 0.16, *p* > 0.05). The variance explained in the outcome (i.e., L2 performance) was rather high (*R*^2^ = 0.89) indicating robust predictive power of the structural bifactor ESEM model. Although some would argue that the high value in *R*^2^ is a result of overfitted model, this is, indeed, expected given the inherent complexity of the bifactor ESEM solution (e.g., the number of parameters estimated is larger than in standard CFA models).

**TABLE 3:** Bifactor CFA, bifactor ESEM, and structural bifactor ESEM model fit indices for the SDT-L2.

Model	χ^2^	*df*	SRMR	RMSEA (Low 90/Hi 90%)	CFI	TLI
Bifactor CFA	334.516*	125	0.07	0.09 (0.08/0.10)	0.91	0.88
Bifactor ESEM	204.798*	94	0.03	0.07 (0.06/0.09)	0.96	0.91
Structural bifactor ESEM	265.722*	131	0.05	0.07 (0.06/0.09)	0.94	0.90

*p < 0.05: error terms of two items on intrinsic motivation have been correlated.

## Discussion

The present study aimed at providing precise information about the factorial structure of the domain-specific scale, the SDT-L2, by adopting the advanced framework of ESEM that allows for the combination of EFA, CFA, and SEM into a single assessment (Marsh et al., 2009; Morin et al., 2013, 2016; Alamer and Marsh, 2022). The researcher used the recently suggested model specification within the ESEM framework, that is the bifactor ESEM, to allow for the assessment of global factors (Howard et al., 2018). The current study demonstrated the usefulness of the bifactor ESEM for the language learning domain, and especially for L2 psychological scales by showing its application in testing the dimensionality of the SDT-L2 scale. In line with previous research, it appeared that bifactor ESEM outperformed the bifactor CFA in the goodness-of-fit, owning to its flexibility in the analysis. Therefore, the theoretical framework of SDT has been supported in the bifactor ESEM model because of the inclusion of the two overarching constructs (i.e., autonomous motivation and controlled motivation), in which the items loaded properly on these general factors while also loaded on their specific factors (Ryan and Deci, 2017). This preposition was not possible to be accounted for in the EFA or the standard CFA models (Guay et al., 2015). Importantly, the findings of the present study contradict the high correlation between intrinsic regulation and external regulation reported in Ardasheva et al. (2012), and showed that the bifactor ESEM can be a more realistic representation of the measurement.

In addition, the present study has shown that bifactor ESEM can be used to provide evidence for convergent and discriminant validity from a new empirical perspective. That is, convergent validity was achieved by showing that items were strongly loaded on their hypothesized factors, even though they were allowed to cross-load on the other factors. Discriminant validity was achieved by showing that items presumed to load on one factor did not load substantially on the others in the ESEM solution, and if cross-loaded they presented weaker loading than on their presumed factor. Moreover, the present study was uniquely able to provide information on the predictive validity of the SDT-L2 scale within the framework of ESEM. By turning the bifactor ESEM into a structural bifactor ESEM model we were able to gain precise and high-quality results of the role of the global constructs (i.e., autonomous motivation and controlled motivation) in predicting the outcome. Because of the flexibility of the bifactor ESEM model the explained variance was quite high (i.e., 89% of the variance in the outcome was explained by the variables in the bifactor ESEM model). In line with SDT literature, it was found that autonomous motivation positively and meaningfully related to an increase in L2 achievement, while controlled motivation failed to predict the outcome (Oga-Baldwin and Nakata, 2017; Alamer and Lee, 2019, 2021; Dincer et al., 2019; Noels et al., 2019).

Although the present study used an advanced method (i.e., bifactor ESEM) to evaluate the multidimensionality of SDT in the language learning domain, it has some limitations. First, the study used a relatively moderate sample size from one socio-cultural context, thus we would like to see replication of this study on other contexts using larger sample sizes. In addition, the present study relied on cross-sectional data. Future studies could benefit from using longitudinal data to account for the invariance of the measure over time.

Despite these limitations, the findings of the current research confirm the validity of the SDT-L2 scale to be used to assess students’ L2 motivation from the SDT perspective. The study is the first to employ the bifactor ESEM in language learning domain and I hope that researchers apply it in their psychometric research. Researchers are encouraged to use the ESEM (and bifactor ESEM) method for construct validation purposes. Going beyond the traditional CFA should help researchers assess their scales from a novel and more appropriate perspective.

## Data Availability Statement

The raw data supporting the conclusions of this article will be made available by the authors, without undue reservation.

## Ethics Statement

The studies involving human participants were reviewed and approved by the Imam Mohammed Ibn Saudi Islamic University. The patients/participants provided their written informed consent to participate in this study.

## Author Contributions

The author confirms being the sole contributor of this work and has approved it for publication.

## Conflict of Interest

The author declares that the research was conducted in the absence of any commercial or financial relationships that could be construed as a potential conflict of interest.

## Publisher’s Note

All claims expressed in this article are solely those of the authors and do not necessarily represent those of their affiliated organizations, or those of the publisher, the editors and the reviewers. Any product that may be evaluated in this article, or claim that may be made by its manufacturer, is not guaranteed or endorsed by the publisher.
