# Supplementary material for: Construct Validation of Self-Determination Theory in Second Language Scale: The Bifactor Exploratory Structural Equation Modeling Approach
Source: Front Psychol. 2021 Sep 30;12:732016. doi: 10.3389/fpsyg.2021.732016 (PMC8514625; doi:10.3389/fpsyg.2021.732016)
Supplement: Supplementary file 1 [file Data_Sheet_1.pdf]

Appendix

*Self-Determination Theory of Second Language Scale (SDT-L2) **

Why are you learning English?

Item
Autonomous motivation
**Intrinsic orientation**
Because I enjoy learning English
Because of the pleasure I get when hear and read English
For the satisfaction I feel when I speak and write in English
For the enjoyment I experience when I achieve a new goal in English learning
Because learning English is a fun activity in and of itself
**Identified orientation**
Because learning English is important for my personal growth
Because learning English can open new opportunities and possibilities for me
For the value it holds in my self-development
Because learning English is important for my current and future studies
Because learning English allows me to read and hear English-based materials that are necessary for my personal success
Controlled motivation
**Introjected orientation**
Because I would feel guilty if I didn't understand English
Because I would feel ashamed if I'm not successful in English learning like my friend(s)/family
Because people around me (the teacher/peers/parents) expect me to learn English
Because people around me (the teacher/peers/parents) would think I'm a failure if I didn't speak English
Because I feel pressured by the people around me (the teacher/peers/parents) to learn English
**External orientation**
Because I want to get a prestigious job that requires English proficiency
Because I want to get better marks in the English course
Because English is just a required course that I want to pass
Because I don't want to fail the final exam in the English course
Because there will be negative consequences if I fail to learn English

***(Alamer, 2021a,b)**

Reference

Alamer, A. (2021a). Basic psychological needs, motivational orientations, effort, and vocabulary knowledge: A comprehensive model. *Studies in Second Language Acquisition*. https://doi.org/10.1017/S027226312100005X.

Alamer, A. (2021b) Construct validation of self-determination theory in second language scale: The bifactor exploratory structural equation modeling approach. *Frontiers in Psychology*. https://doi.org/10.3389/fpsyg.2021.732016
